# A miRNAs panel promotes the proliferation and invasion of colorectal cancer cells by targeting GABBR1

**DOI:** 10.1002/cam4.760

**Published:** 2016-05-27

**Authors:** Yang Longqiu, Luo Pengcheng, Fei Xuejie, Zhang Peng

**Affiliations:** ^1^ Department of AnesthesiologyHuangshi Central HospitalAffiliated Hospital of Hubei Polytechnic UniversityEdong Healthcare GroupHuangshi435000China; ^2^ Department of Urology SurgeryHuangshi Central HospitalAffiliated Hospital of Hubei Polytechnic UniversityEdong Healthcare GroupHuangshi435000China; ^3^ Department of Intensive Care UnitShuguang Hospital Affiliated with Shanghai University of Traditional Chinese MedicineShanghai200021China; ^4^ Department of OncologyThe Center Hospital of Zaozhuang Mining GroupZaozhuang277000China

**Keywords:** Colorectal cancer, GABBR1, invasion, miR‐106a/b, miR‐17, miR‐20a/b, proliferation

## Abstract

MicroRNAs (miRNAs) have been implicated in the regulation of colorectal cancer. Despite the expression of miR‐17‐92 cluster in cancer has been gradually revealed, the role of each individual miRNAs in colorectal cancer still remains unclear. We studied the impact of miR‐106a/b, miR‐20a/b, and miR‐17 of miR‐17‐92 cluster on colorectal cancer cells. Real‐time quantitative polymerase chain reactions (RT‐PCR) were used to test these five miRNAs expression in colorectal cancer cell line HCT116. 3‐(4,5‐dimethyl‐2‐thiazolyl)‐2,5‐diphenyl‐2‐H‐tetrazolium bromide (MTT) assays, Bromodeoxyuridine (BrdU), and Transwell invasion assays were used to explore the effects of these five miRNAs in colorectal cancer cells. Luciferase reporter assay, RT‐PCR, and western blotting were performed to validate the interaction of these five miRNAs with the gamma‐amino‐butyric acid type B receptor 1(GABBR1). We found that these five miRNAs were significantly upregulated in colorectal cancer samples compared with normal tissues. Forced expression of these five miRNAs significantly promoted HCT116 and HT‐29 cells proliferation and invasion. We further found that these five miRNAs function as oncogenes in colorectal cancer by specifically binding to the 3‐untranslated regions (3′UTR) of GABBR1.Furthermore, inhibition of GABBR1 could mimic the function of miRNAs in HCT116 cells, while overexpression of GABBR1 blocked the function of miRNAs‐promoted proliferation and invasion. In conclusion, miR‐106a/b, miR‐20a/b, and miR‐17 contribute to the proliferation and invasion of colorectal cancer by targeting their common target gene, GABBR1, and played a critical role in the proliferation and invasion of colorectal cancer.

## Introduction

Colorectal cancer is one of the most common malignant tumors which contribute to the fifth cause of tumor death rates in China 1, 2. The incidence of colorectal cancer is nearly 5% and the 5‐year survival rate ranges from 40% to 60% 3. Many protein‐coding genes are involved in the regulation of proliferation of colorectal cancer cells. Stromal cell‐derived factor‐1 (SDF‐1) has been reported to be associated with metastases of human colorectal cancer and local tumor progression 4. PKC Beta II is also a tumor suppressor in colorectal cancer cells survival 5. Downregulation of Rpt4 reduced colorectal cancer growth 6. These studies showed that many protein‐coding genes can regulate the growth of colorectal cancer. Additionally, there are many noncoding RNAs, such as miRNAs, which can also participate in the regulation of the colorectal cancer cells' initiation and development.

miRNAs are a family of small noncoding RNAs that regulate gene expression by binding to the 3‐untranslated regions (3′UTR) of target mRNAs 7. Increasing number of studies suggested that miRNAs affect the progression, pathogenesis classification, diagnosis, and prognosis of cancer 8, 9, 10, 11.Recently, increased miRNAs have been implicated in the digestive diseases 12, 13, 14.Several studies have found that miR‐21, miR‐17, miR‐31, miR‐15, and others were critically involved in the pathogenesis of colorectal cancer 15, 16, 17, 18. miR‐17‐92 cluster consisted of six miRNAs (miR‐17, miR‐18a, miR‐19a, miR‐20a, miR‐19b, miR‐92a) and two paralogs (miR‐106b and miR‐106a). Some of them have been validated to be upregulated in colorectal cancer tissues 19. However, the role of each individual miRNA in colorectal cancer has not been fully investigated. It is well known that miRNAs regulate gene expression by inhibiting protein translation or by triggering degradation of the target mRNAs. Although some individual targets have been reported for miR‐106b 20, it has been hypothesized that these miRNAs function through cooperative suppression of targets because they share the same seed region. However, whether this miRNA family can participate in the growth of colorectal cancer and the downstream target genes needs to be investigated.

GABBR1, also known as GABABR1, is a 7‐transmembrane receptor, which is mapped to chromosome 6p21.3 within the HLA class I region close to the HLA‐F gene 21. GABBR1 and GABBR2 receptors are two subunits of GABA_B_ receptor which play a pivotal role in hepatocellular carcinoma and pancreatic carcinoma 22, 23, 24. However, as a novel and important epigenetic regulator, the upstream regulator of GABBR1 still remains the subject of ongoing studies.

To better understand the mechanism of the miRNAs cluster regulation, we studied the functional properties of these miRNAs in colorectal cancer cells. Our results showed that miR‐106a/b, miR‐20a/b, and miR‐17 could enhance colorectal cancer cell proliferation and invasion. These miRNAs could all directly target GABBR1 mRNA 3′UTR. We found that activation of GABBR1 signaling could repress the proliferation and invasion of HCT116 cells. On the contrary, downregulation of GABBR1 promoted the proliferation and invasion of HCT116 cells .Further, we found that overexpression of GABBR1 significantly restored the function of miRNAs on regulating proliferation and invasion. This study uncovered the mechanism of proliferation and invasion regulation via this miRNAs family / GABBR1 signaling pathway and provided an insight into further therapeutic targets of colorectal cancer cells.

## Materials and Methods

### Cell culture

Human colorectal cancer cell lines, HCT116 cells, were obtained from the American Type Culture Collection. HCT116 cells were cultured in 1640 medium (Gibco, Grand Island, NY, USA) supplemented with 10% fetal bovine serum (FBS, Gibco) and 100 U/mL of penicillin sodium (Invitrogen, Life Technologies, California, USA) and 100 mg/mL of streptomycin sulfate (Invitrogen, Life Technologies). HCT116 cells were incubated at 37°C in 5% CO2 atmosphere.

### RNA isolation and RT‐PCR

Total RNA, including miRNA, was extracted using RNAiso Reagentreagent (Takara, Japan) according to the manufacturer's instruction. GABBR1 mRNA level was tested using SYBR PrimeScript RT‐PCR Kit (Takara) with the following primers: GABBR1 forward primer, 5′‐GAGGACGTGAATAGCCGCAG‐3′ and GABBR1 reverse primer, 5′‐CTGGATCACACTTGCTGTCGT‐3′. GAPDH forward primer, 5′‐CTGGGCTACACTGAGCACC‐3′, reverse primer: 5′‐AAGTGGTCGTTGAGGGCAATG‐3′. For miRNA analysis, the stem‐loop RT primer and the RT‐PCR primers for each individual miRNAs were purchased from Ribobio (Guangzhou, China). The relative expression of each individual miRNAs was normalized to that of the internal control U6.

### miRNA and siRNA transient transfections

Cells were plated 24 h prior to transfection with miRNAs mimics or siRNAs in a 6‐well dish. All of the miRNA mimics were designed by and purchased from Ribobio (Guangzhou, China). Small interfering RNA (siRNA) was designed and purchased from Gene Pharma (Shanghai, China). Transient transfections were carried out using Lipofectamine 2000 (Invitrogen), according to the reagent protocol.

### MTT assay

Cells were plated at a density of 5 × 10^3^ cells per well in 96‐well plates at 37°C in 5% CO_2_ in incubator. MTT reagent was added to each well at the indicated time periods. After 4 h of incubation, the medium was removed and 150 *μ*L DMSO/well was added. The absorbance was measured according to the manufacturer's instructions.

### BrdU assay

1.5 × 10^3^ cells/well cells were cultured in 96‐well dish in the medium with a final concentration of 10μM BrdU. After incubation for 3 h, cells were fixed by 4% paraformaldehyde (PFA) for 15 min, then washed by PBS solution and treated with 0.2 TrtonX‐100, after which they were further washed and treated with DNase (Tiangen, China) for 20 min at room temperature. Then washed the cells with PBS and added BrdU primary antibody (Abcam, Cambridge, MA, USA) and incubated the cells at 4°C for 12 h. After washing the cell with PBS, cells were incubated with secondary antibody for 60 min. Washed the cells with PBS and detected the BrdU positive cells by fluorescence microscope.

### Western blotting and antibodies

Anti‐GAPDH and anti‐GABBR1 were purchased from Abcam. For western blotting, cells were lysed with 1 × SDS‐PAGE loading buffer and then transferred onto PVDF membranes (Millipore). The antibody dilutions were 1:2000 for anti‐GAPDH and 1:1000 for anti‐GABBR1.

### Cell invasion

Cell invasion assay was carried out using transwell inserts with 8.0 *μ*m pores (Corning 3422) in 24‐well plates. Transwell chamber was coated with matrigel (BD Bioscience; 50 mg/mL; 1:8) at 37°C for 4 h. Cells were cultured in the upper chambers of a transwell (Corning) with serum‐free medium. Medium in the bottom chamber contained 10% FBS. Cells (5 × 10^5^) were seeded on the upper chamber and incubated for about 15 h. A quantity of 4% PFA was added to fix the cells on the membrane for 10 min. Cells were stained with Hoechst 33342 away from light for 5 min and then washed with PBS three times. Finally, 24‐well plates were observed under a fluorescence microscope and the number of cells with the blue‐fluorescence were counted from 4–5 randomly microscopic filed.

### Luciferase reporter assay

The GABBR1 3′‐UTR segment containing the target site for five‐miRNAs panel was amplified from genomic DNA and inserted into the PGL3. Complementary seed region was mutated using the Quik Change mutagenesis Kit (Stratagene, Santa Clara, CA, USA). The constructs were confirmed using DNA sequencing. 1 × 10^4^ HCT116 cells were seeded in 24‐well and cotransfected with 100 ng wild‐type or mutant reporter construct, 20 nmol/L miRNA and 10 ng Renilla using Lipofectamine 2000. The activity of firefly luciferase and Renilla was tested at 48 h post transfection using a dual‐luciferase assay system (Progema, Madison, WI, USA) according to the manufacturer's instructions.

### Different expression analysis

We collected data from the TCGA (The Cancer Genome Atlas) database of cancer and normal samples. The cancer type we used is colorectal cancer. The level of miRNA‐seq data available in TCGA is of level 3. The number of cancer samples we collected was 1104 and the number of normal samples was 106. We used the value of RPKM for the downstream analysis. To make the data stable, we first filtered the top 5 % and bottom 5 % samples. Then according to the density of the expression value in cancer and normal samples, we filtered some samples based on our experience. Then we performed the Wilcox‐test in R (version:3.2.2, Vienna, Austria) between normal and cancer samples and got the *P*‐value of the two states in each miRNA.

### Statistical analysis

Statistical analyses were performed with SPSS version 17.0 software (IBM SPSS Statistics, Armonk, NY, USA). Student's *t*‐test was used to determine the level of significance of differences between two groups. Values were presented as the mean ± SD.

## Results

### miR‐106a/b, miR‐20a/b, miR‐17 upregulated in colorectal cancer

**Figure 1:**
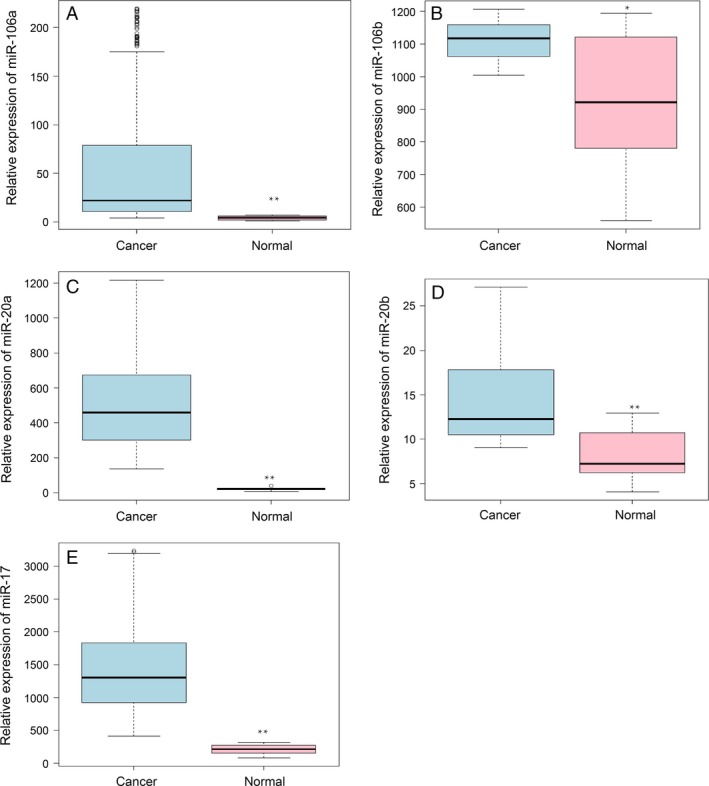
miR‐106a/b, miR‐20a/b, and miR‐17 were upregulated in the colorectal cancer compared with normal tissues. The expression level of the miR‐106a (A), miR‐106b (B), miR‐20a (C), miR‐20b (D), miR‐17 (E) between colorectal cancer (light blue) and normal (pink) samples in The Cancer Genome Atlas database. The data has been filtered. The y‐axis is the FPKM value of the miRNA. *P < 0.05, **P < 0.01.

In order to find out the main miRNAs which may have correlation with the colorectal cancer, we collected level 3 data from the TCGA database of cancer and normal samples. We suppose that in colorectal cancer and normal samples some miRNAs showed abnormal expression. After data filtering of the miRNA‐seq, we use Wilcoxon test to calculate *P*‐value of the miRNA expression in two states. Then we found the five miRNAs significantly upregulated in cancer samples compared with normal tissues: miR‐106a (Fig. 1A), miR‐106b (Fig. 1B), miR‐20a (Fig. 1C), miR‐20b (Fig. 1D), miR‐17 (Fig. 1E).

We noticed that these five miRNAs shared similar seed sequence (Fig. S1A). As for correlation of these five miRNAs, we first filtered the miRNA‐seq data of colorectal cancer in the TCGA database, then extracted the expression of the five miRNAs and adopted data normalization. After that, we drew the scatter plot of them at the lower left in Figure S1B and wrote the correlation coefficient on the upper right (Fig. S1B). The correlation coefficient among miRNAs is larger than 0.3, which means they are moderately correlated.

### miR‐106a/b, miR‐20a/b, and miR‐17 promoted the proliferation and invasion of colorectal cancer cell

**Figure 2:**
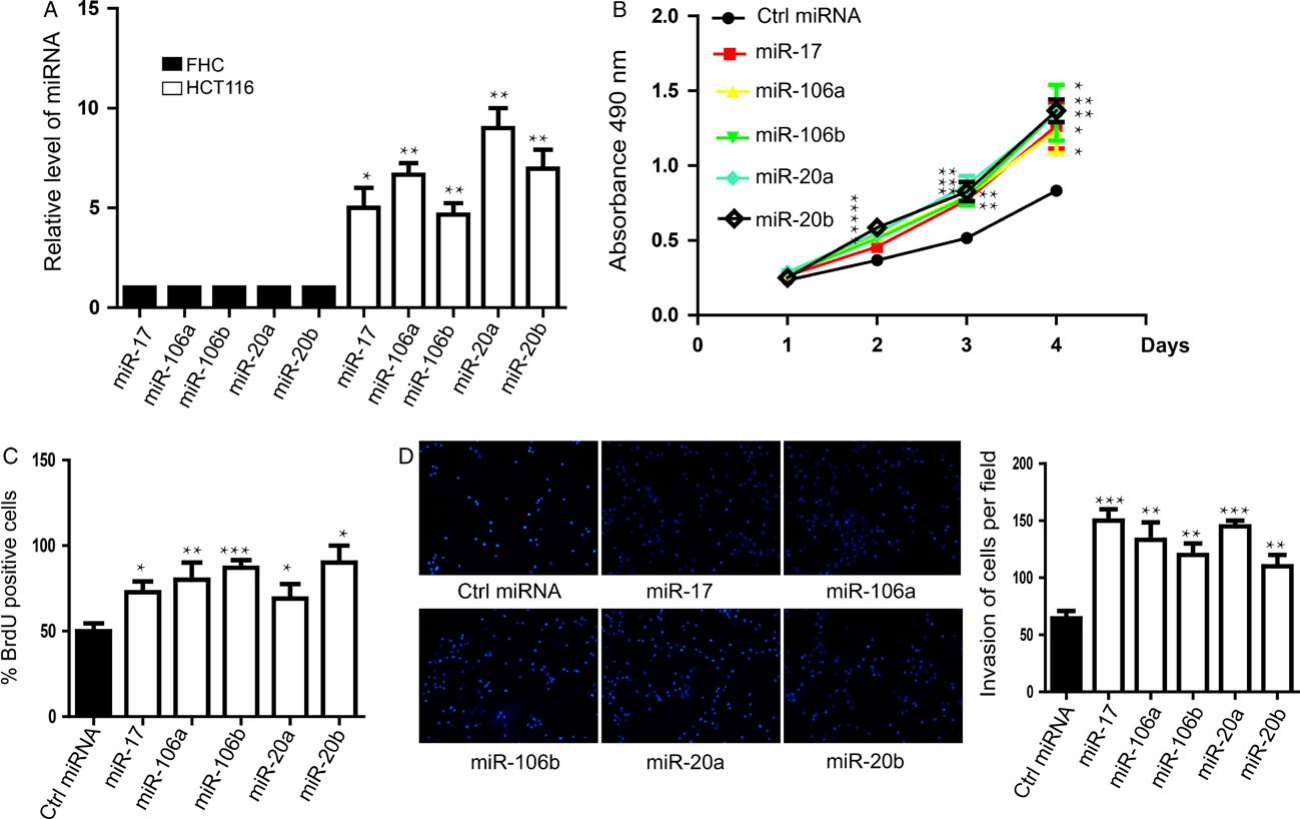
miR‐106a/b, miR‐20a/b, and miR‐17 promoted the proliferation and invasion of HCT116 cells. (A) The expressions of these five miRNAs in HCT116 cells were significantly upregulated compared with the control. Data shown are means ± SD (*n* = 5). *P < 0.05, **P < 0.01. (B) MTT assay was performed to determine cell proliferation with the treatment of these miRNAs mimics. Data shown are means ± SD (*n* = 5). *P < 0.05, **P < 0.01. (C) Bromodeoxyuridine assay was carried out to further confirm miRNAs‐promoted proliferation. Data shown are means ± SD (*n* = 3). *P < 0.05, **P < 0.01, ***P < 0.001. (D) Transwell assay was performed to measure the invasive capacity of HCT116 cells with the treatment of these miRNAs mimics. Right penal showed the statistics of the invasion. Data shown are means ± SD (*n* = 4). **P < 0.01, ***P < 0.001.

RT‐PCR was performed to detect the expression of a five‐miRNAs panel in colorectal cancer cell HCT116. We found that all of these miRNAs were significantly upregulated in HCT116 compared with fetal colon cell line FHC cells which is the normal colorectal mucosa cell line (Fig. 2A). These findings drew us to further investigate their biological roles in colorectal cancer. MTT assay was conducted to measure the growth rate of HCT116. We observed that overexpression of these miRNAs respectively promoted the proliferation of HCT116 cells (Fig 2B). Additionally, these miRNAs also promoted the proliferation of HT‐29 cell which is another kind of colorectal cancer cell line (Fig S2A). It is quite surprising that overexpression of each single miRNA could promote HCT116 or HT‐29 cell proliferation. To further validate our results, BrdU assay was carried out to quantify the cell growth rate and the data showed the similar results as the MTT assay (Figs. 2C, S2B). Next, the role of five‐miRNAs panel in colorectal cancer cell invasion, respectively, was examined using transwell assay. The results showed that these miRNAs could promote HCT116 or HT‐29 cells invasion (Fig. 2D, Fig. S2C).

### GABBR1 is a target gene of miR‐106a/b, miR‐20a/b, and miR‐17

**Figure 3:**
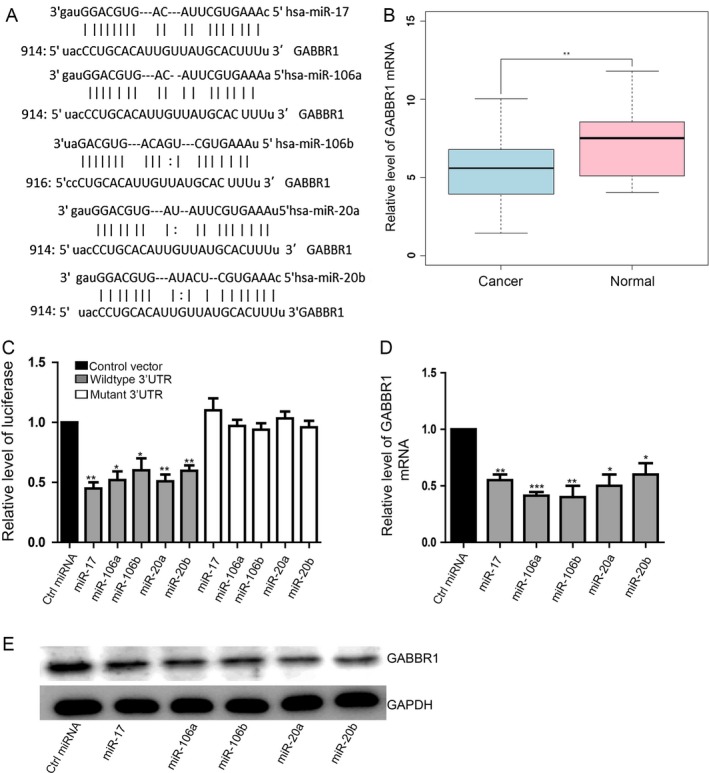
GABBR1 is a target gene of miR‐106a/b, miR‐20a/b, and miR‐17. (A) The predictive binding site between GABBR1 and these five miRNAs. Data shown are means ± SD (*n* = 3). **P < 0.01, ***P < 0.001. (B) mRNA‐seq data of colorectal cancer and normal samples were collected from The Cancer Genome Atlas. The expression level of the GABBR1 between colorectal cancer (light blue) and normal (pink) samples was shown. The y‐axis is the log2 RSEM (RNA‐Seq by Expectation Maximization) value of the gene. **P < 0.01. (C) The luciferase activity of wild‐type‐3′UTR showed a significant decrease as a result of these five miRNAs while the luciferase activity of mutant‐3′UTR was not altered. Data shown are means ± SD (*n* = 4). *P < 0.05, **P < 0.01. (D) Overexpression of these five miRNAs significantly suppressed GABBR1 expression on mRNA level. Data shown are means ± SD (*n* = 3). *P < 0.05, **P < 0.01, ***P < 0.005. (E) Representative pictures showed that overexpression of these five miRNAs significantly suppressed GABBR1 expression on protein level. 3′UTR, 3‐untranslated regions.

miRNAs has been demonstrated to regulate cell proliferation and invasion by suppressing gene expression in a sequence‐specific manner 9. miRWalk, Targetscan, and Miranda predicted that GABBR1 might be their potential common target gene. Bioinformatic algorithms showed that conserved 3′UTR sequences of GABBR1 complementary to seed region at the 5′ end of these five miRNAs active strand (Fig. 3A). The expression of GABBR1 gene in normal samples is higher than the tumor samples; however, the expression level of five miRNAs was opposite (Fig. 1A). Bioinformatic algorithms showed that conserved 3'UTR sequences of GABBR1 is complementary with the seed region at the 5'end of these five miRNAs strand (Fig. 3A). Luciferase reporter gene assay was performed to validate our hypothesis. These five miRNAs mimics significantly inhibited GABBR1 3′UTR reporter luciferase activity by more than 30% and their mutation in the binding site blocked this inhibition (Fig. 3C). Then HCT116 was respectively transfected with these five miRNA mimics and we found that GABBR1 expression showed a significant decrease on both mRNA (Fig. 3D) and protein level (Fig. 3E). We also found that GABBR1 mRNA (Fig. S3A) and protein level (Fig. S3B) could be downregulated by these miRNAs in HT‐29 cells .Our results suggested that this five‐miRNAs panel specifically bound the predicated translation repressor site on GABBR1 and inhibited GABBR1 expression in HCT116.

### GABBR1 are critically involved in the proliferation and invasion of HCT116

**Figure 4:**
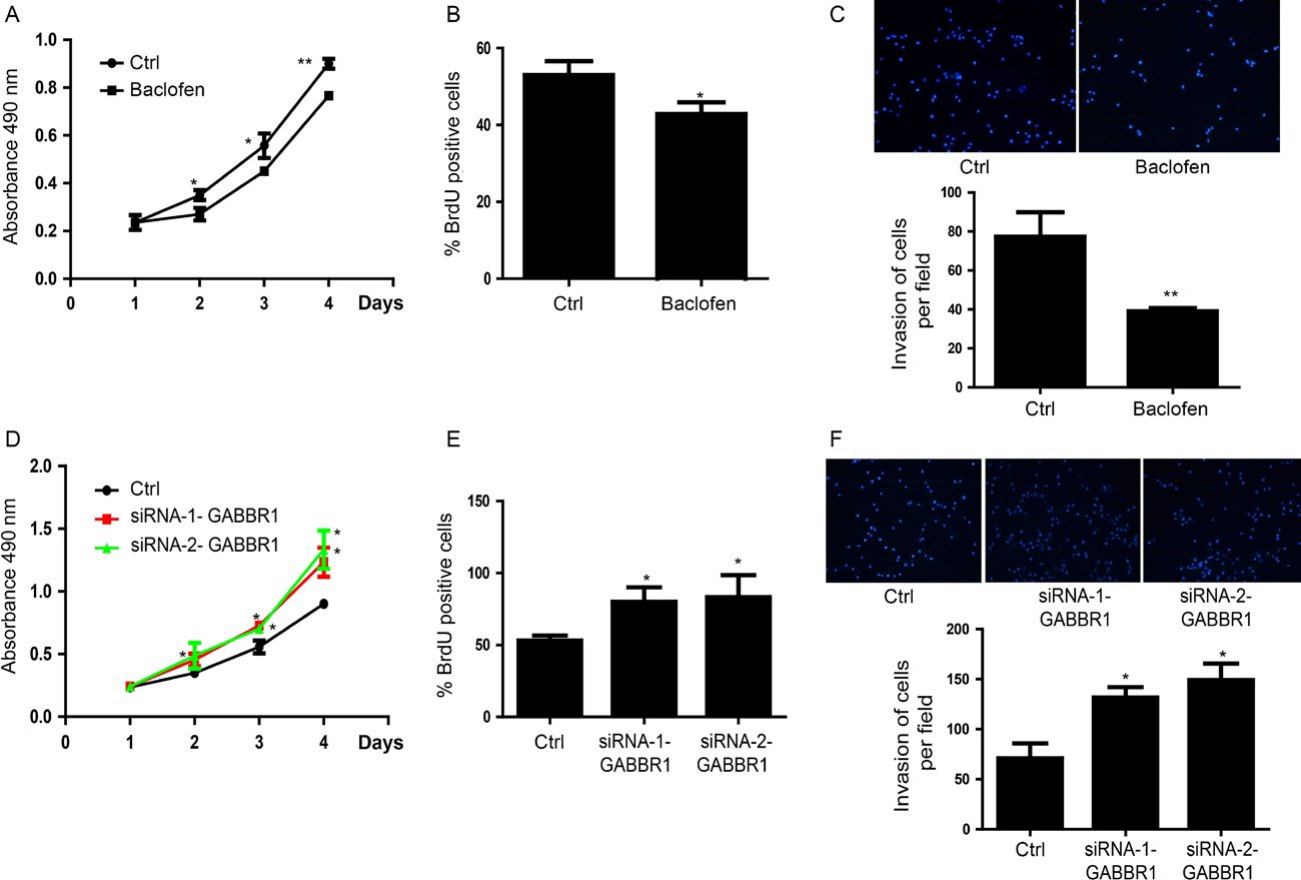
GABBR1 is critically involved in the proliferation and invasion of HCT116 cells. (A) Baclofen inhibited HCT116 cells proliferation detected by MTT assay. Data shown are means ± SD (*n* = 4). *P < 0.05, **P < 0.01. (B) BrdU assay showed that baclofen could repress cell proliferation. Data shown are means ± SD (*n* = 5),*P < 0.05. (C) Baclofen could significantly inhibit HCT116 cells invasion. Data shown are means ± SD (*n* = 3) **P < 0.01. (D) Inhibition of GABBR1 by two different GABBR1 specific siRNA sequences enhanced HCT116 proliferation detected by MTT assay. Data shown are means ± SD (*n* = 5). *P < 0.05. (E). BrdU assay showed that siRNA‐mediated inhibition of GABBR1 promoted HCT116 proliferation, which is consistent with MTT assay. Data shown are means ± SD (*n* = 3),*P < 0.05. (F) siRNA‐mediated inhibition of GABBR1 obviously promoted HCT116 invasion. Right penal showed the statistics of the invasion. Data shown are means ± SD (*n* = 5),*P < 0.05. BrdU, Bromodeoxyuridine.

Next, we hypothesized that GABBR1 was also involved in the proliferation and invasion of colorectal cancer cell. HCT116 cells were used to be the model in vitro and treated with baclofen, a selective GABBR1 receptor agonist, to activate GABA signaling. The data suggested that baclofen had a negative influence on cell proliferation and invasion. HCT116 cells were treated with baclofen that is a selective GABBR1 receptor agonist to activate GABA signaling could inhibit cell proliferation (Fig. 4A and B). Besides, cells invasion was also suppressed as a result of GABA signaling activation (Fig. 4C). Moreover, we wanted to find that whether siRNA‐mediated silencing of GABBR1 mimicked the effects of these miRNAs in HCT116 cells. First, we detected the effect of siRNAs (Fig. S4A).Then we found that inhibition of GABBR1 promoted cell proliferation (Fig. 4D and E) and invasion (Fig. 4F).Two different GABBR1‐specific siRNA sequences suggested similar results.

### Overexpression of GABBR1 blocked the function of miR‐106a/b, miR‐20a/b and miR‐17 to promote proliferation and invasion

**Figure 5:**
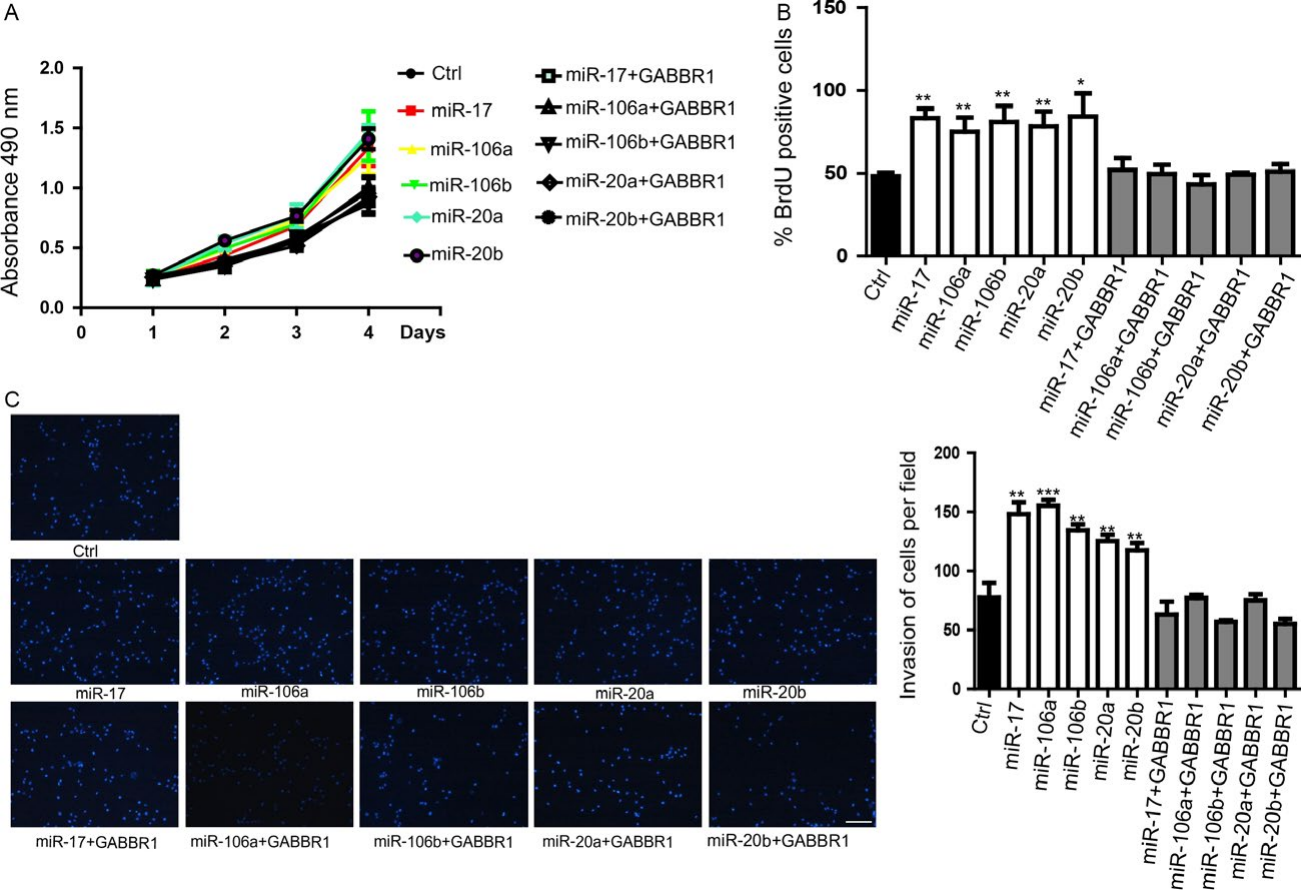
Overexpression of GABBR1 blocked the function of miR‐106a/b, miR‐20a/b, and miR‐17 on promoting proliferation and invasion. (A) MTT assay suggested that forced expression of GABBR1 could block miRNA‐promoted cell proliferation. Data shown are means ± SD (*n* = 3),(B) BrdU also indicated that GABBR1 overexpression could block miRNA‐promoted cell proliferation. Data shown are means ± SD (*n* = 3),*P < 0.05, **P < 0.01. (C). Transwell assay showed that GABBR1 overexpression could rescue miRNA‐promted cell invasion. Right penal showed the statistics of the invasion. Data shown are means ± SD (*n* = 4), **P < 0.01, ***P < 0.001. BrdU, Bromodeoxyuridine.

To determine whether this five‐miRNAs panel promoted cell proliferation and invasion by inhibiting GABBR1 expression levels, we carried out rescue experiments by introducing exogenous GABBR1 in these miRNAs overexpressed HCT116 cells. Firstly, HCT116 cells were infected with a lentiviral vector that overexpressed GABBR1 without 3′UTR. Subsequently, cells were transfected with these five miRNAs respectively. We detected that overexpressed GABBR1 significantly restored the GABBR1 level which was repressed by overexpression of miRNAs (Fig. S5A). The results of MTT assay (Fig. 5A) and BrdU assay (Fig. 5B) indicated that overexpression of GABBR1 could counteract miRNAs‐induced proliferation. Moreover, the invasive capacity of HCT116 cells was restored by overexpression of GABBR1 in the cells overexpressed with the five miRNAs, respectively. The data suggested that forced expression of GABBR1 was able to block miRNAs‐promoted invasion (Fig. 5C).

## Discussion

In summary, we uncovered that the miR‐106a/b, miR‐21a/b, miR‐17 which were higher expressed in the colorectal cancer tissues than in normal tissues downregulated the GABBR1 expression to influence the proliferation and invasion of colorectal cancer. Overexpression of these miRNAs repressed the proliferation of HCT116 cells. Overexpression or activation of GABBR1 significantly inhibited the colorectal cancer cell, HCT116 cells proliferation. Further, downregulation of GABBR1 could repress the cell proliferation which was the same as the overexpression of the miRNAs, respectively. Additionally, GABBR1 restored the capacity of proliferation and invasion of HCT116 cells which transfected with the miRNAs, respectively. These results might provide new insight into the critical roles of GABBR1 signaling axis which regulated by miR‐106a/b, miR‐21a/b, and miR‐17 in colorectal cancer and laid the foundation for clinical treatment of colorectal cancer in the future.

miRNAs have been reported to be the critical regulator of initiation and development of many kinds of cancers. Many miRNAs are involved in the regulation of cell proliferation in many kinds of physiological processes 25, 26, 27, 28.In colorectal cancer, miRNAs have been the potential biomarkers of colorectal cancer 29, 30.miR‐106a/b, miR‐20a/b, miR‐17 are the members of miR‐17‐92 cluster and have been demonstrated to participate into the signaling pathways to regulate the initiation and development of cancers 31. miR‐106b promoted colorectal cancer cell migration and invasion by directly targeting DLC1 20.miR‐106a also has been reported to play an oncogenic role in pancreatic cancer 32. Upregulation of miR‐20a level acts as a potential biomarker of aggressive progression and poor prognosis of cervical cancer 33. miR‐20b has been shown to be aberrantly expressed in several tumor types and might serve as a potential molecular marker for the prognosis of gastric cancer 34. c‐Myc/ miR‐17 feedback loop regulates metastasis and invasion of hepatocellular carcinoma 35. In this study, we found that miR‐106a/b, miR‐20a/b, and miR‐17 were highly expressed in the colorectal cancer tissues than in normal tissues. Overexpression of these miRNAs, respectively, in the HT116 cells significantly promoted the proliferation. These results showed that miR‐106a/b, miR‐20a/b, and miR‐17 were critically involved in the regulation of colorectal cancer cells. Additionally, these miRNAs also might be the potential biomarkers of colorectal cancer.

GABB receptors are members of the G‐protein coupled receptor (GPCR) superfamily which include metabotropic glutamate, opioid, and olfactory receptors 36. GABBR mediated the transmission which regulates the excitation threshold of the local circuitry such as subiculum 37. GABB receptors have been the important targets of` neurological diseases' treatment. Additionally, recent studies showed that GABB receptor also has been the potential target for tumor therapy 38. GABB receptor has been detected in human hepatocellular carcinoma (HCC) cells. Baclofen could inhibit human HCC cell growth 39. GABBR also has been reported to be upregulated in thyroid cancer tissues when compared with the corresponding normal tissues 40. In our study, we found that miR‐106a/b, miR‐20a/b, and miR‐17 directly targeted the same site of the GABBR1 3′UTR in colorectal cancer. The expression level of GABBR1 also was negatively correlated with that of miRNAs in cancer tissues. Knockdown of the GABBR1 significantly repressed the proliferation of HCT116 cells, which was similar with the function of miR‐106a/b, miR‐20a/b, and miR‐17. On the contrary, overexpression of GABBR1 promoted the proliferation of HCT116 cells. Additionally, overexpression of GABBR1 restored the capacity of proliferation that was repressed by miRNAs. These studies showed that GABBR1 significantly regulates the proliferation and invasion and might be the potential therapeutic target. So, the agonist of GABBR1 signaling might be the potential effective drug for colorectal cancer therapy. Additionally, we found that miR‐106a/b, miR‐20a/b, and miR‐17 targeted GABBR1 to regulate the GABB signaling during the colorectal cancer cells growth. These results also suggested that GABBR1 formed the signaling axis with these miRNAs to regulate the proliferation and invasion of colorectal cancer cells. Because the regulation of miRNAs targeting downstream genes is in vivo progress, the miRNAs might also be the potential therapeutic target which is more suitable than antagonist of GABBR1 to some extent. Previous study showed that histone H2Ax‐dependent GABAA receptor regulated stem cell proliferation 41. GABAA receptor could also modulate the Chk1/p53 signaling pathway to influence the propofol‐induced apoptosis and proliferation inhibition of rENSCs 42. But less study showed the mechanism of GABB signaling on regulating proliferation. Our next unanswered question is how the pathway of miR‐106a/b, miR‐20a/b, and miR‐17 regulate GABBR1 to influence the colorectal cancer proliferation and invasion. We will perform further studies to investigate the downstream pathway of GABBR1 in colorectal cancer. Our study not only showed the mechanism of proliferation regulation via this miRNAs family/GABBR1 signaling pathway but also suggested some new therapeutic targets of miRNAs and GABBR1 signaling for further treatment.

## Conflict of Interest

None declared.

## Supporting information


**Figure S1.** miRNAs showed significantly relationship between each others.

**Figure S2.** miR‐106a/b, miR‐20a/b and miR‐17 promoted the proliferation and invasion of HT‐29 cells.

**Figure S3.** miRNAs downregulate the GABBR1 expression in HT‐29 cells.

**Figure S4.** Detection of effect of GABBR1 siRNA.

**Figure S5.** Overexpressed GABBR1 restored the GABBR1 level which was repressed by overexpression of miRNAs.
