# Supplementary material for: A miRNAs panel promotes the proliferation and invasion of colorectal cancer cells by targeting GABBR1
Source: Cancer Med. 2016 May 27;5(8):2022–31. doi: 10.1002/cam4.760 (PMC4884921; doi:10.1002/cam4.760)

A

3' -gauggacgugacAUUCGUGAAAc- 5' hsa-miR-17

3' -gauggacgugauAUUCGUGAAAu -5' hsa-miR-20a

3'- gauggacgugauACUCGUGAAAc- 5' hsa-miR-20b

3'- gauggacgugacauUCGUGAAAa -5' hsa-miR-106a

3' -uagacgugacagUCGUGAAAu -5' hsa-miR-106b

B

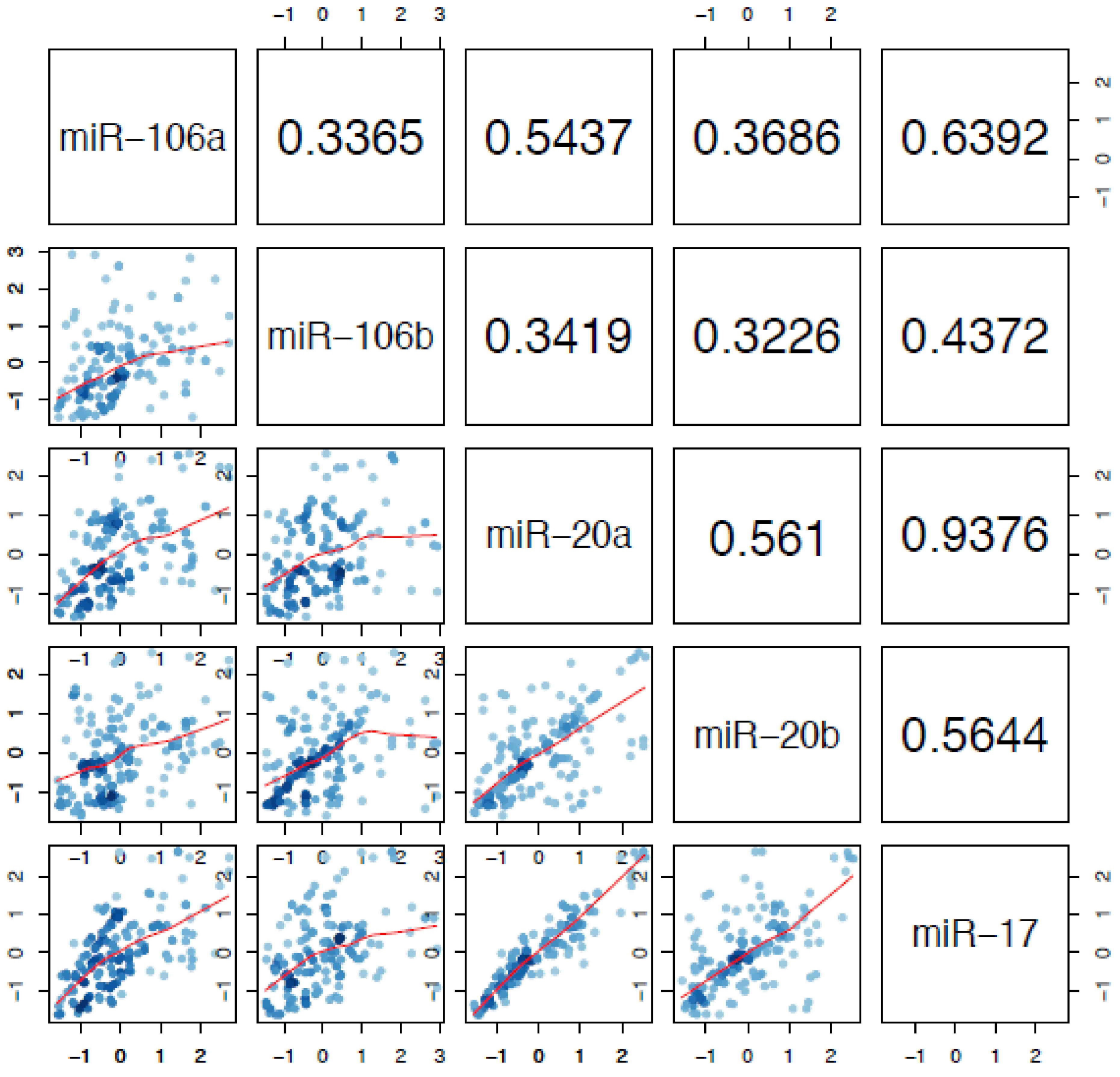

Supplement: Supplementary file 1 — Figure S1. miRNAs showed significantly relationship between each others. [file CAM4-5-2022-s001.pdf]
