# Supplementary figures and images for: A miRNAs panel promotes the proliferation and invasion of colorectal cancer cells by targeting GABBR1
Source: Cancer Med. 2016 May 27;5(8):2022–31. doi: 10.1002/cam4.760 (PMC4884921; doi:10.1002/cam4.760)

A

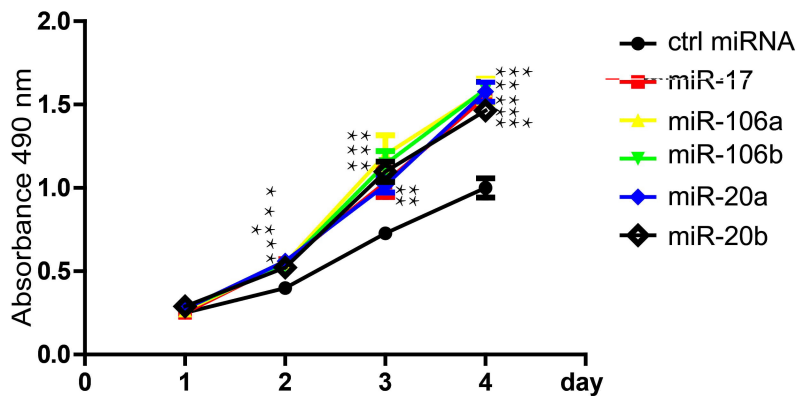

B

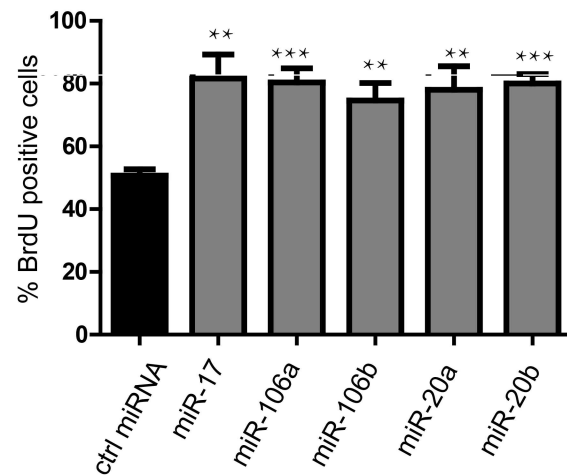

C

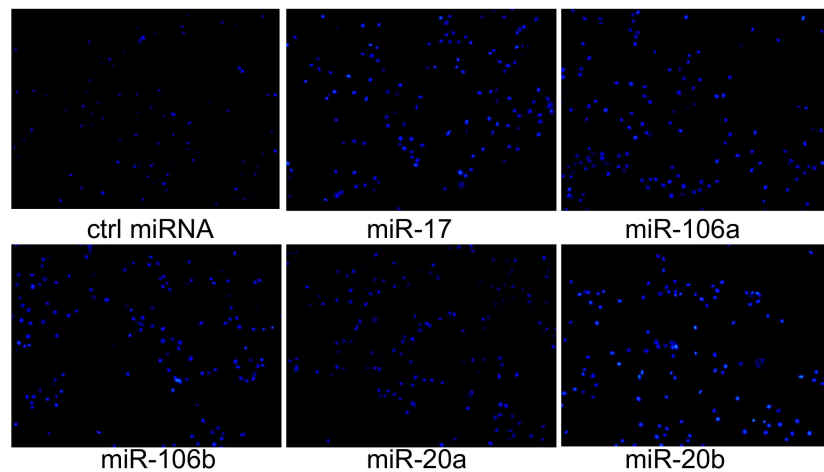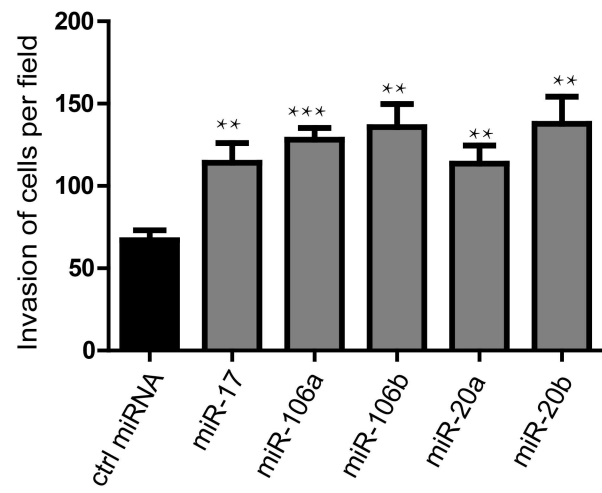

Supplement: Supplementary file 2 — Figure S2. miR‐106a/b, miR‐20a/b and miR‐17 promoted the proliferation and invasion of HT‐29 cells. [file CAM4-5-2022-s002.pdf]

**A**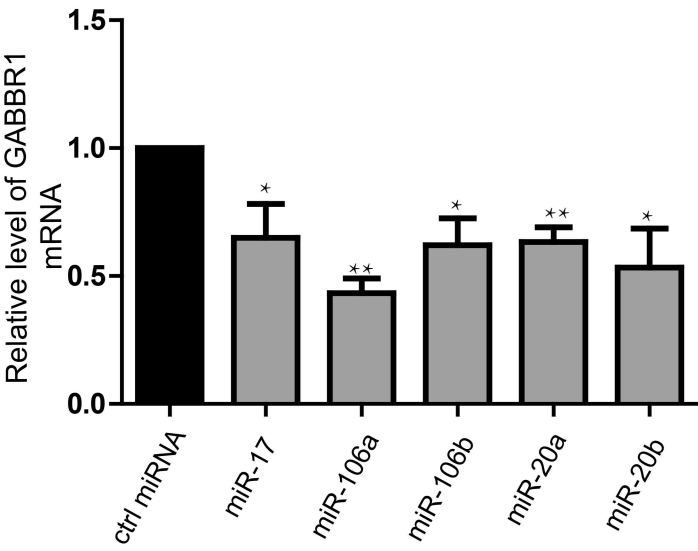**B**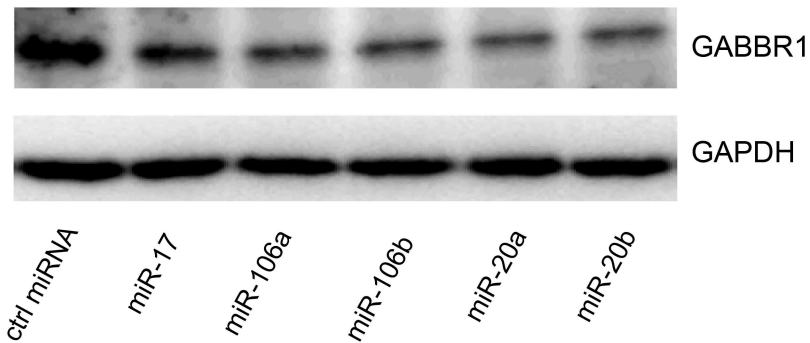

Supplement: Supplementary file 3 — Figure S3. miRNAs downregulate the GABBR1 expression in HT‐29 cells. [file CAM4-5-2022-s003.pdf]

**A**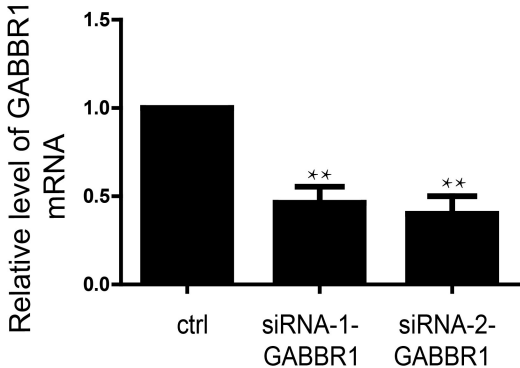

Supplement: Supplementary file 4 — Figure S4. Detection of effect of GABBR1 siRNA. [file CAM4-5-2022-s004.pdf]

**A**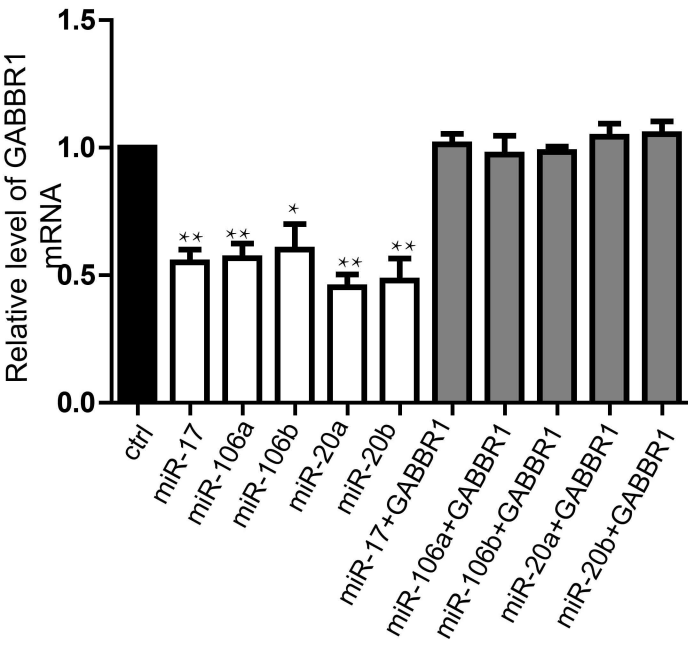

Supplement: Supplementary file 5 — Figure S5. Overexpressed GABBR1 restored the GABBR1 level which was repressed by overexpression of miRNAs. [file CAM4-5-2022-s005.pdf]
